# A five-safes approach to a secure and scalable genomics data repository

**DOI:** 10.1016/j.isci.2023.106546

**Published:** 2023-03-31

**Authors:** Chih Chuan Shih, Jieqi Chen, Ai Shan Lee, Nicolas Bertin, Maxime Hebrard, Chiea Chuen Khor, Zheng Li, Joanna Hui Juan Tan, Wee Yang Meah, Su Qin Peh, Shi Qi Mok, Kar Seng Sim, Jianjun Liu, Ling Wang, Eleanor Wong, Jingmei Li, Aung Tin, Ching-Yu Cheng, Chew-Kiat Heng, Jian-Min Yuan, Woon-Puay Koh, Seang Mei Saw, Yechiel Friedlander, Xueling Sim, Jin Fang Chai, Yap Seng Chong, Sonia Davila, Liuh Ling Goh, Eng Sing Lee, Tien Yin Wong, Neerja Karnani, Khai Pang Leong, Khung Keong Yeo, John C. Chambers, Su Chi Lim, Rick Siow Mong Goh, Patrick Tan, Rajkumar Dorajoo

**Affiliations:** 1 Genome Institute of Singapore (GIS), Agency for Science, Technology and Research (A∗STAR), 60 Biopolis Street, Genome #02-01, Singapore 138672, Republic of Singapore; 2 Singapore Eye Research Institute, Singapore National Eye Centre, Singapore, Republic of Singapore; 3 Yong Loo Lin School of Medicine, National University of Singapore, Singapore, Republic of Singapore; 4 Duke-NUS Medical School, Singapore, Republic of Singapore; 5 Singapore National Eye Centre, Singapore, Republic of Singapore; 6 Department of Paediatrics, Yong Loo Lin School of Medicine, National University of Singapore, Singapore, Republic of Singapore; 7 Khoo Teck Puat - National University Children’s Medical Institute, National University Health System, Singapore, Republic of Singapore; 8 Department of Epidemiology, Graduate School of Public Health, University of Pittsburgh, Pittsburgh, PA, USA; 9 UPMC Hillman Cancer Center, University of Pittsburgh, Pittsburgh, PA, USA; 10 Healthy Longevity Translational Research Programme, Yong Loo Lin School of Medicine, National University of Singapore, Singapore, Republic of Singapore; 11 Singapore Institute for Clinical Sciences (SICS), Agency for Science, Technology and Research (A∗STAR), Brenner Centre for Molecular Medicine, 30 Medical Drive, Singapore 117609, Republic of Singapore; 12 Saw Swee Hock School of Public Health, National University of Singapore, Singapore, Republic of Singapore; 13 Braun School of Public Health, Hebrew University of Jerusalem, Jerusalem, Israel; 14 Precision Health Research, Singapore, Republic of Singapore; 15 SingHealth Duke-NUS Institute of Precision Medicine, Singapore, Republic of Singapore; 16 Molecular Diagnostic Laboratory, Tan Tock Seng Hospital, Singapore, Republic of Singapore; 17 Clinical Research Unit, National Healthcare Group Polyclinics, Singapore, Republic of Singapore; 18 Lee Kong Chian School of Medicine, Nanyang Technological University, Singapore, Republic of Singapore; 19 Bioinformatics Institute (BII), Agency for Science, Technology and Research (A∗STAR), 30 Biopolis Street, Matrix #07-01, Singapore 138671, Republic of Singapore; 20 Personalised Medicine Service, Tan Tock Seng Hospital, Singapore, Republic of Singapore; 21 Department of Cardiology, National Heart Centre Singapore, Singapore, Republic of Singapore; 22 School of Public Health, Imperial College London, London, UK; 23 Clinical Research Unit, Khoo Teck Puat Hospital, Singapore, Republic of Singapore; 24 Diabetes Centre, Admiralty Medical Centre, Singapore, Republic of Singapore; 25 Institute of High Performance Computing (IHPC), Agency for Science, Technology and Research (A∗STAR), 1 Fusionopolis Way, #16-16 Connexis (North Tower), Singapore 138632, Republic of Singapore; 26 Cancer Science Institute of Singapore, National University of Singapore, Singapore, Republic of Singapore

**Keywords:** Genomics, Data encryption, Data storage representation

## Abstract

Genomic researchers increasingly utilize commercial cloud service providers (CSPs) to manage data and analytics needs. CSPs allow researchers to grow Information Technology (IT) infrastructure on demand to overcome bottlenecks when combining large datasets. However, without adequate security controls, the risk of unauthorized access may be higher for data stored on the cloud. Additionally, regulators are mandating data access patterns and specific security protocols for the storage and use of genomic data. While CSP provides tools for security and regulatory compliance, building the necessary controls required for cloud solutions is not trivial. Research Assets Provisioning and Tracking Online Repository (RAPTOR) by the Genome Institute of Singapore is a cloud-native genomics data repository and analytics platform that implements a “five-safes” framework to provide security and governance controls to data contributors and users, leveraging CSP for sharing and analysis of genomic datasets without the risk of security breaches or running afoul of regulations.

## Highlights

• RAPTOR implements a “five-safes” framework for security and governance of genomic data
• RAPTOR provides policy-compliant access to cloud computing for sensitive genetic data
• We demonstrated proof of concept for federated analysis using GA4GH protocols
• RAPTOR is an option as a trusted custodian of national-scale genomic data

## Graphical abstract

**Figure:**
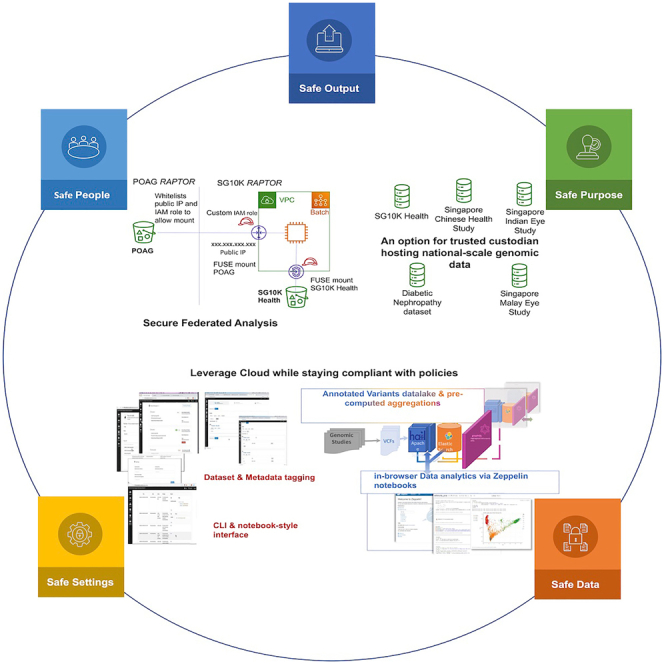

## Introduction

Data footprint for genomics projects has increased rapidly. The UK Biobank, for example, holds about 11 petabytes of genomic data, which are projected to grow above 40 petabytes by 2025.^1^ To effectively store and process data at petabyte scale requires significant information technology (IT) capacity, operated by experienced specialists. These capacities are often out of reach for small-to-medium size companies and academic labs. There is also the added issue of effectively sharing large-scale data with collaborators. To transfer 1 petabyte of data across a network, which allows actual sustained throughput (not line speed) of 1024Mbps, will take more than 90 days. If data are moved through the public internet, the transfer time is estimated to be at least 5 times longer.^2^

Public cloud computing platforms, such as Amazon Web Services (AWS), Google Cloud Platform (GCP), and Microsoft Azure, provide feasible ways around these constraints. Commercial clouds provide elastic and scalable IT resources (i.e., servers and storage) allowing users to grow their IT infrastructure in tandem with data generation without loss in reliability, availability, or performance.^3^^,^^4^^,^^5^ Operators can also adjust the scale and subsequent costs of cloud-based IT operations on demand, to match different project phases. In contrast, operators building on-premise data centers must build in sufficient capacity to take on the peak load of the project and not the most common load level. Therefore, an on-premise system operator will have to bear the cost of maintaining the entire system designed for peak usage, even during lull periods when most of the servers are idle. In addition, once data are available on the cloud, collaboration and sharing can be achieved by having users run analytics within the same “cloud region” where the data reside, effectively side-stepping the challenge of moving large data across networks. The National Human Genome Research Institute (NHGRI) Analysis Visualization and Informatics Lab-space (AnVIL) project, for example, leverages GCP to host and share more than 3 petabytes of genomic data.^6^ For analysis, AnVIL provides its users with the ability to work directly on cloud by integrating with various analytics platforms, including Galaxy, Juypter, and Dockstore. These platforms enable users to bring computation to the data repository, effectively “inverting the model of data sharing”.^6^ However, unrestricted use of cloud computing can introduce significant security risks. Without well-designed access controls, data placed on cloud can potentially be accessed from anywhere and by anyone with internet access. Active data on cloud may spend considerable time moving through storage and servers shared with many other users, allowing data to be silently replicated many times over while in flight. Furthermore, cloud data sharing requires setting up an endpoint which is accessible from the internet. As evidenced by remote attacks through OpenSSH with GNU Bash, this may create opportunities for malicious actors who can either break in using forged credentials or vulnerabilities in the software used to host the data.^7^

There have been several initiatives to address these security concerns of genomic data. The Global Alliance for Genomics and Health (GA4GH) has developed protocols, tools, and policies for responsible sharing of genomics data.^8^ In particular, the GA4GH Data Security workstream has delivered a data security infrastructure policy, which outlines recommendations for securing IT infrastructure used for genomic and clinical data.^9^ The Authentication and Authorization Infrastructure guide additionally provides a comprehensive framework for cloud users to safely authenticate users and assign authorizations for data use.^10^ The Ministry of Health of Singapore has also issued a HealthTech Instruction Manual (HIM), providing instructions for IT and data governance.^11^ These include standard procedures and algorithms for data encryption, configuration of network partition and data access points, and management and securing user accounts.^12^

Major cloud service providers (CSPs) already supply many tools needed to build a secure platform following best practices and compliance to regulations.^13^^,^^14^^,^^15^ Groups such as the National COVID Cohort (N3C) by the National Center for Advancing Translational Sciences^16^ have built virtual data enclaves that leverage scale and elasticity of CSP while enforcing strict access controls, such as blocking all data egress from the enclave. Such data enclave implementations may be too restrictive for facilitating other genomic research and collaborations. For example, researchers may prefer to use a customized analytics environment that comes with a wide range of tools and reference data from the internet. On an enclave such as N3C, users will have to work with analytics environment that comes with the platform. Other platforms such as AnVIL, while providing extensibility and ease of integration with other platforms and excellent flexibility for researchers, may not be aligned with specific national regulations on how genomic data should be protected. For example, human whole-genome data from public hospitals have been classified as “Restricted Sensitive” under the Singapore Government’s data classification framework. As such, building a large data platform on CSP remains a complex task requiring deep IT expertise and knowledge of relevant regulations that may not be available in an academic genomics lab.^17^

**Figure 1:**
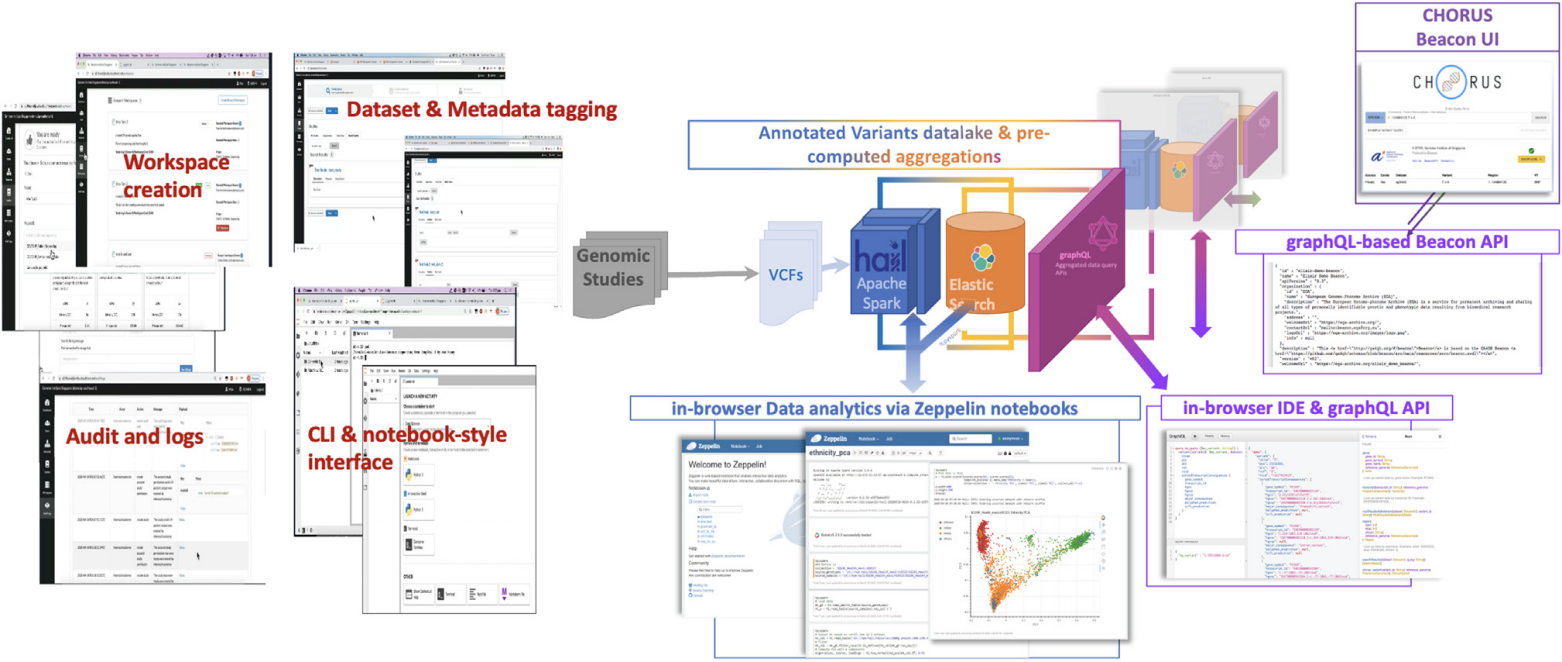
RAPTOR Overview and the modes of data analysis supported RAPTOR provides access via Standalone Linux machines with sudo access, Juypter Notebooks, and EMR Cluster with pre-configured Hail tools.

The Research Assets Provisioning and Tracking Online Repository (RAPTOR) was developed to fill this niche. RAPTOR is a serverless, cloud-native, genomics data repository and analytics platform that strives to balance flexibility for data use with IT security and regulatory compliance (Figure 1). Our key compliance measures include enforcing encryption at rest and in flight with key separation (using pre-approved algorithms only), stringent egress controls, and data that must be hosted within Singapore’s jurisdiction (applicable to CSP). RAPTOR is hosted on AWS, Singapore region*.* Through the use of a “5-safes” framework, RAPTOR provides researchers the ability to build their own analytics environment to leverage elasticity and scalability of cloud computing for large-scale data analysis, without having to worry about IT security and regulatory compliance. With data-in-place analytics, RAPTOR also circumvents the challenge of transferring large datasets across public networks and allows strong accountability of data usage.

## Results

RAPTOR is designed to be foundationally secure and embedded with all elements essential for data governance. Security and data governance strategies and procedures are considered and designed into the platform using a “5-Safes” framework—safe purpose, safe people, safe settings, safe data, and safe output.

### Safe purpose

Safe purpose refers to measures adopted to ensure data contributors’ control of data deposited with RAPTOR*.* Users who wish to access a dataset must submit an access request on the platform. Mandatory fields of this request include their proposal and the duration of their access. Users who wish to access datasets beyond the original deadline must submit an extension request through the platform.

It is mandatory to provide at least one data access committee (DAC) contact when depositing data onto RAPTOR, and RAPTOR will forward any access request to the relevant DAC. After evaluation, the DAC has the option to approve or deny the access request using RAPTOR’s data management console. The DAC may also choose to grant access to specific subsets of files or allow access with modified parameters. For instance, a DAC may choose to modify certain data access expiry dates for specific sub-datasets on the same console.

RAPTOR hosts data on AWS S3*.* The hosting buckets are configured to block all access except those coming through a specific S3 endpoint*.* An S3 endpoint is analogous to a proxy for a webserver, providing an interface for the bucket to interact with the outside world without any direct connection. Access policies can be applied to the endpoint to govern traffic to the bucket. For example, in the case of allowing read access from a specific Elastic Computing Cloud (EC2) instance, when data users request to work on a dataset, a customized policy is generated on the fly based on permissions granted by the DAC.

Once access has been granted, users can analyze the dataset using RAPTOR’s Analytics Workspace. This is an on-demand, dedicated virtual network where all interactions by a user with the chosen dataset can occur. The Analytics Workspace comes in three flavors: single-node AWS EC2 virtual machine instance, elastic spark cluster, and high-performance computing cluster. Through this design, RAPTOR automates the provisioning of selected data, creation and configuration of virtual machines, and the enforcement of security policies, on one computational resource.

Within the EC2 instance, users have full administrator rights by default and can install tools directly from the internet. To save operating costs, workspaces can be shut down when not in use, with the virtual server retaining its mount points, tools, and environment. Only the terminate command will destroy the workspace completely. Users have the option of exporting their virtual machine, the Amazon Machine Image (AMI), to be shared with collaborators. AMIs flagged for export will be reviewed by RAPTOR administrators to ensure the image is safe and does not contain malware data. Administrators also will work with relevant DAC to ensure the custom image does not violate terms of use. Second, RAPTOR’s elastic spark cluster invokes AWS Elastic MapReduce Service to provision an Apache SPARK cluster (https://spark.apache.org/) with a Zeppelin notebook (https://zeppelin.apache.org/) serving as the front end. This workspace also comes with common genomic analysis tools, such as Hail (https://hail.is/) pre-installed to facilitate data analytics. Third, within the high-performance computing cluster, users can create a dedicated Linux computing cluster complete with SLURM scheduler (https://www.schedmd.com/) and a share storage for processing data requiring heavy computational demands.

Workspaces do not create local copies of datasets. The operating system mount points read data directly from where selected datasets are housed on RAPTOR, thereby allowing users to sidestep the issue of moving large data files across the network. RAPTOR uses AWS FSX to provision a Lustre file system (https://www.lustre.org/) for both runtime scratch and analysis outputs. Outputs and results are flushed into S3 for persistent storage when the workspace is shut down. AWS service endpoints are used to route function calls to native AWS services. This allows the workspace to function even if all external network access is disabled. Similar to data on S3, data on scratch and output staging are encrypted with advanced encryption standard, 256 bits (AES256) with key separation.

Importantly, the DAC can modify the conditions of data being shared even after RAPTOR has granted user access. For instance, DACs may revoke a user’s permission to modify the original dataset while still allowing the user to access data for specific analysis. Additionally, the DAC may stop all access immediately with a kill switch, i.e., the mounted drive containing the selected dataset will immediately disconnect from the analytics workspace. Under safe usage, data contributors are thus able to have effective and direct control of datasets housed on RAPTOR.

Mechanisms are also in place to protect against “insider-attacks” from malicious users with valid RAPTOR credentials. Access to resources within RAPTOR, including data on S3, are restricted using policies for both network addresses and identity and access management (IAM) role of the calling application programming interface (API). Upon a user starting a workspace, RAPTOR creates a new IAM role unique to the user and the request. This newly created role will be attached to all API calls from its attached workspace. Policies guarding data access will allow role access only to data, which 1) were specifically requested during workspace creation and 2) has had access granted by the relevant DAC. Attempts by a user to access any other data (assuming the user has gained insider information on paths on S3) will be rejected by RAPTOR’s service endpoints. This created role will be destroyed upon workspace termination. Additionally, all analytic workspaces are segregated by subnets, and traffic between workspace subnets is blocked. This prevents malicious users attempting to steal session credentials by running metadata queries on another concurrently running workspace.

Table S1 provides key security differentiators between RAPTOR, the National Center for Advancing Translational Sciences (NCATS) N3C data enclave, and NHGRI AnVIL. Notably, RAPTOR administrators are out of loop regarding data access requests. Rather, representatives from the appropriate DACs will approve or reject requests using the RAPTOR console, and RAPTOR adminstrators only provide technical support to DAC representatives.

### Safe user

RAPTOR ensures all users within the system are properly validated and does not allow anonymized access. As part of the user registration process, RAPTOR administrators verify the identity of the applicant. This typically involves running checks with the applicant’s institute or collaborator.

User management and authentication controls in RAPTOR are handled by AWS Cognito due to its compliance with key security standards including ISO 27001, HIPPA BAA, and Multi-Tiered Cloud Security (MTCS).^18^^,^^19^ RAPTOR does not store user credentials. All user records are encrypted both at rest and in transit. A 2-factor authentication is mandatory for all users for better protection, which also discourages users from sharing accounts. Additionally, Cognito allows integration with major identity providers, including active directory, and supports protocols including OAuth2, security assertion markup language (SAML), and OpenID Connect,^20^ facilitating future integration with systems that conform to GA4GH authentication and authorization infrastructure.^21^

RAPTOR’s safe user protocols can also extend to the platform’s core administration and development team. For example, in the case of Singapore, developers and system administrators working on RAPTOR must receive security clearance from Singapore’s Ministry of Home Affairs to work with restricted data. These measures ensure that data deposited in RAPTOR will only be accessed by approved individuals.

### Safe settings

**Figure 2:**
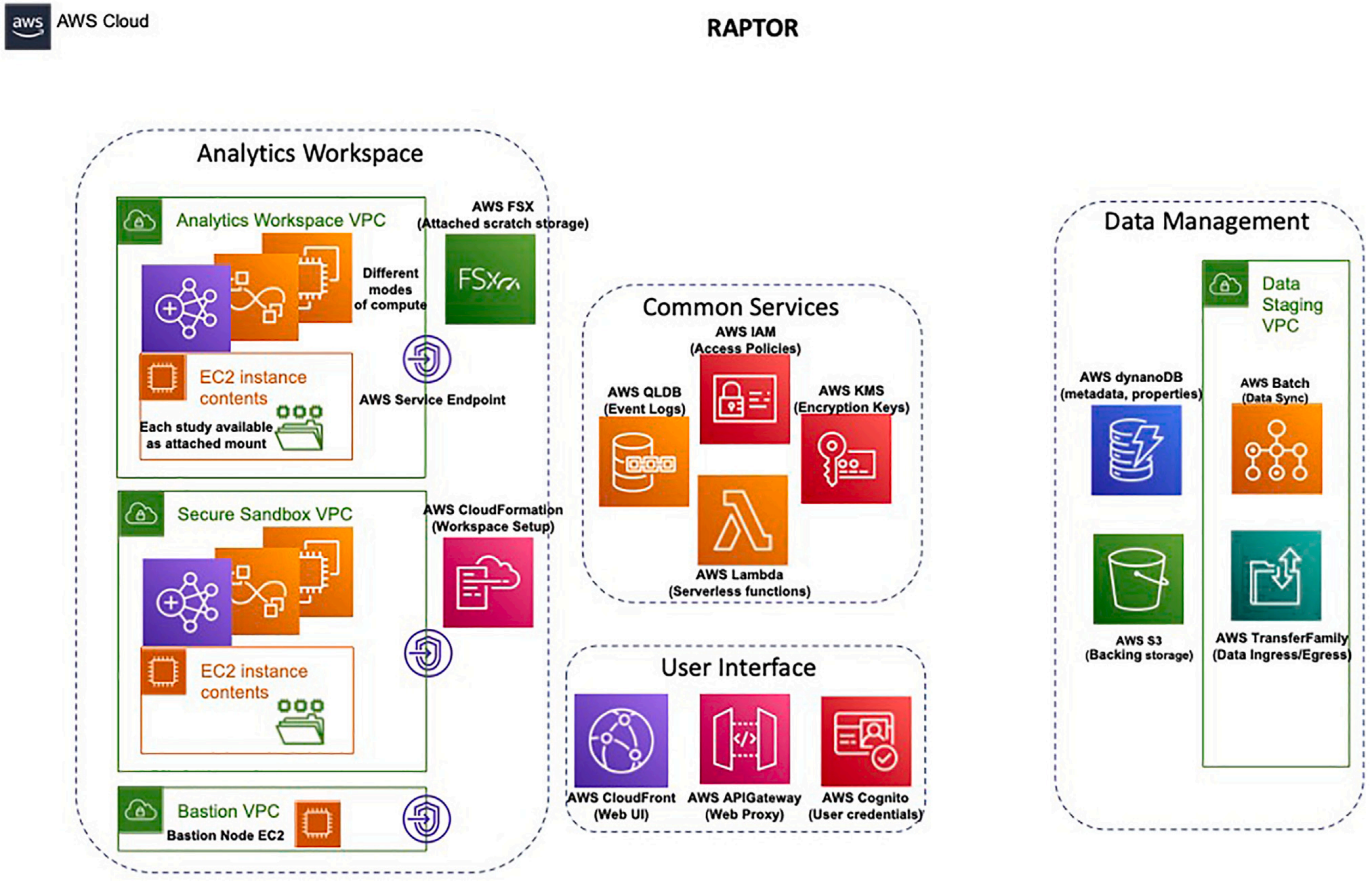
As a serverless application, RAPTOR is composed of native AWS services integrated together with Lambda functions User Interfaces are composed of CloudFront hosting graphical user interfaces made with Java scripts. User authentications are managed with Cognito. Hosted datasets sit on S3 (with automated tiering) while all metadata are stored on DynamoDB. Data-staging activities are managed using S3 Batch. Data ingress and egress are managed through TransferFamily. The Analytics workspace relies on FSX to provide scratch storage, and depending on the mode of compute, either EC2, Elastic Map Reduce or Parallel Cluster will provide computing power. Data access from the nodes is regulated by Service Endpoints. All permissions and authorisations are managed using IAM. Encryption keys used by S3, EBS, and DynamoDB are stored within KMS. All RAPTOR activities are written into AWS QLDB.

RAPTOR employs a multi-layer strategy to protect hosted data against unauthorized access, starting with the host infrastructure. RAPTOR is a serverless, cloud-native application that leverages CSP with the Platform-*as*-a-Service (PaaS) model. RAPTOR’s features are constructed from scripts and functions by hosting CSP’s native services (Figure 2). For example, RAPTOR’s graphical user interface is built from a set of Java scripts hosted on AWS CloudFront, meaning that RAPTOR does not manage or operate any servers. Notably, AWS is among CSP adopted to host public services for the Singaporean government^22^ and was one of the first to attain MTCS level 3 (highest level) under SS584:2020, a security standard designed by Infocomm Media Development Authority (IMDA) Singapore,^23^ which is required for CSP to host data and services for the Singapore government. Thus, adopting AWS in RAPTOR for application and data security allows us to provide stringent operating procedures for infrastructure security.

Data encryption provides an added layer of data protection. RAPTOR encrypts all data with symmetric AES256. This extends to data stored on interim server stores within the analytics workspace, dataset metadata, and search indexes. To guard against accidental leakage and to provide additional protection against malicious actors, data encryption keys are stored in a separate system, the AWS key management system (KMS). RAPTOR leverages AWS Parameters Store to encrypt key IDs to prevent accidental leakage of key identifiers when invoking routines. All connections between the user’s computer and RAPTOR are encrypted with SSH2, rivest cipher 4 (RC4), or trasport layer security (TLS) version no older than 1.2.

RAPTOR further provides enhanced data protection for data requiring egress restrictions with the Secure Analytics Workspace (Secure Sandbox). This is a locked-down version of a regular analytics workspace. It provides complete network isolation (no internet), blocking all data egress. A specially provisioned bastion node using Remote Desktop Protocol (RDP) with copy and print redirection disabled is the only way for users to access a Secure Analytics Workspace. Bastion nodes can further restrict its access to whitelisted IPs and subnets.

To validate the effectiveness of RAPTOR’s security measures, RAPTOR undergoes penetration tests and vulnerability assessment by Council of Registered Ethical Security Testers (CREST)-certified assessors at least once every twelve months, to coincide with our platform revision cycle. During the annual assessment, RAPTOR will be assessed against well-known exploits (published Common Vulnerabilities and Exposure [CVEs]) and potential weakness in any of RAPTOR’s system dependencies. Our current release cycle deploys a major revision every 10 months, and we engage a CREST assessor to ensure that there has been no inadvertent weakening of our security posture in the new release. In addition, between a major release, the team will run open worldwide application security project (OSWAP) vulnerability and dependency scanners every three months to ensure RAPTOR stays current with respect to security patches. Critical security issues from Computer Emergency Response Team (CERT) alerts are addressed as soon as possible (for example, zero-day exploits).

Beyond the direct security measures*,* RAPTOR has an extensive event logging system, which tracks all actions performed on datasets, including each time it comes up during a search, when a user submits an access request, and every instance the dataset is provisioned to a workspace. RAPTOR’s logs provide network (transmission control protocol/internet protocol [TCP/IP])-level granularity and are encrypted to protect against tampering. Logs are kept for at least 12 months and can be made available for evaluations upon request.

Taken collectively, RAPTOR ensures that there are adequate mechanisms to protect hosted data, all system vulnerabilities will be promptly patched, and extensive logs are available to allow reconstruction of events to identify issues or support audits.

### Safe data and safe output

Safe data and safe output refers to RAPTOR’s data protection mechanism for ingress and egress. RAPTOR’s ingress procedure requires the data contributors to deposit their data into a pre-set staging *S3* bucket. Data going into RAPTOR are both screened for malware and checked for authorization from the project’s DAC before they become available for use on RAPTOR*.* Data hosted on RAPTOR cannot be modified without authorization from the data contributor. Data on RAPTOR are transparently spread across multiple AWS S3 storage tiers to optimize between cost and access efficiency. The use of AWS S3 also provides data with 11 9s in data durability and 2 9s in data availability.^24^ For data contributors who desire higher levels of assurance, options such as data immutability, file-level versioning, and cross-region backups are available.

To enable egress protection, RAPTOR allows data contributors to mandate the use of Secure Analytics Workspace for users working on their data. To copy files out of a Secure Analytics Workspace*,* users must submit an egress request on RAPTOR*.* A copy of the data for egress will be made in a bucket. The requestors will not have any access to this bucket, and requestors cannot change the contents of the files to be egressed after submitting a request. RAPTOR will notify the data contributor of the egress request. The data contributor will then have full file-level access to the files RAPTOR had duplicated. The contributor can spin up a “Review workspace” to run content filtering or validation routines on the files submitted. The contributor can subsequently approve the egress request from their data management console, and the requestors can export these approved files. If the egress request consists of several datasets that have been combined, all contributors of the respective datasets are required to approve the egress request before the requestor will be allowed to perform data egress.

### Case study: Secure imputation analysis on RAPTOR

Utilizing population-specific reference panels during imputation can significantly improve the accuracy of detecting low-frequency variants (minor-allele frequency [MAF] <1%) for the relevant study population.^25^^,^^26^^,^^27^^,^^28^ We evaluated the performance of imputing additional genotypes on the Singapore Chinese Health Study (SCHS) dataset (N = 23,600)^28^^,^^29^^,^^30^ and the Asian datasets from the Breast Cancer Association Consortium (BCAC, N = 40,001) using local population-specific reference panels from the SG10K whole-genome sequencing initiative (SG10K Health)^31^^,^^32^ on the RAPTOR platform. Alleles for all SNPs were coded to the forward strand and mapped to HG38. Minimac4 (version 1.0.0) was used to impute variants in the SCHS study using 9,770 local Singaporean population sample reference panels from the SG10K study.^32^ Additional imputation on the same SCHS dataset was performed using Trans-Omics in Precision Medicine (TOPMed)^33^ imputation reference panel (version r2) that includes data from 97,256 reference samples (https://imputation.biodatacatalyst.nhlbi.nih.gov). The quality of imputed SNPs from both analyses was determined by impute r^2^ values; high-quality common SNPs (MAF ≥1%) were those with an impute r^2^ > 0.3, and high-quality rare SNPs (MAF <1%) were those with an impute r^2^ > 0.6.

Figure S1 provides a flowchart detailing sequence of action, from the user’s perspective, including logging into RAPTOR, selecting appropriate datasets, performing analyses, and egressing data. The SG10K data deposited in RAPTOR were flagged as sensitive, and thus the SCHS genotyped data were linked to the SG10K reference panels in the Secure Sandbox. Access to this instance was restricted to a Windows bastion node, and users could only connect to the bastion node with the Windows RDP that has print and clip-board function disabled. Users working on Windows or MacOS will connect to the bastion node with Microsoft’s freely available RDP client, while those working on Linux are recommended to use Remmina, an open-source client for Microsoft RDP. Within the bastion node, users will find Secure Shell (ssh) credentials to access the imputation server, with both the SCHS study and the SG10K panels data available as mount points. After the end of imputation, we submitted an egress request for the folder containing imputed dosage data.

### Ancestry-matched reference panels improve imputation of rare variants

We compared the imputation performance in the SCHS after imputing for additional variants using the Trans-Omics in Precision Medicine (TOPMed)^33^ and local SG10K reference panels using the RAPTOR platform. Expectedly, high-quality common variants (MAF ≥1%) obtained after imputation on the TOPMed and SG10K reference panels were similar (7,236,027 and 7,263,376 biallelic SNPs obtained after TOPMed and SG10K imputations, respectively, Table 1). However, a substantially higher number of high-quality rare variants (MAF <1%) were obtained in the SCHS study through imputation with local SG10K reference panels as compared to the TOPMed imputed data (1,271,426 additional rare SNPs from SG10K imputation procedures, Table 1).

**Table 1:** Numbers of total, common, and rare SNPs obtained after imputation of the SCHS dataset with TOPMed and SG10K imputation panels

	SCHS (N = 23,756)	
	TOPMed Imputation	SG10K Imputation
Total SNPs obtained after imputation	271,221,018	42,602,074
Common SNPs (MAF ≥ 1%) with high imputation quality (r^2^ > 0.3)	7,236,027	7,263,376
Rare SNPs (MAF < 1%) with high imputation quality (r^2^ > 0.6)	9,496,227	10,767,653

### Five-safes framework enabled efficient usage of consortia level data in RAPTOR

BCAC data consisted of South Asian and South-East Asian ancestry subjects genotyped on the Infinium OncoArray (N = 27, 501) and the illumina collaborative oncological gene-environment study (iCOGS) array (N = 12,500). Data were highlighted as sensitive and similarly required mandatory use of the Secure Sandbox, as well as requiring limited usage for only imputation protocols. The BCAC study folder was linked to the SG10K reference panels in the Secure Sandbox, and access to this instance was similarly restricted to a Windows bastion node paired exclusively to the instance running imputation service. Of note, even on a single AWS EC2 instance (r5.8xLarge), this entire work for imputation of the relatively large BCAC dataset (N = 40,001) in a secure setting took only around 20 days (end-to-end) and incurred modest costs of about USD 3,000 worth of AWS utilization, highlighting that the RAPTOR platform enables effective and secure usage of large-scale consortia-level data.

### Remote data retrieval using GA4GH standards

Data integration with other data platforms, such as combining RAPTOR’s genomic data with another repository holding phenotype information, is a key feature of RAPTOR. GA4GH workstreams have defined several standards and APIs to facilitate data federation across different genomic repositories. The first task in any data exchange is to discover and list datasets available in the remote host. This is most effective when performed before initializing user authentication and authorization. Hence, common standards are crucial in enabling two different data sources to exchange “data catalogs” securely without authentication. The GA4GH Discovery workstream’s Beacon V2 standard^34^ provides an efficient mechanism for such activity. We do not anticipate major roadblocks adding this functionality to RAPTOR as the Discovery workstream has provided a working reference for implementation of the Beacon v2 standard. Furthermore, as RAPTOR is serverless, every conceptual “layer” of the application is exposed via APIs and a thin integration layer between the Beacon v2 reference implementation and RAPTOR’s hosting services can be readily incorporated. The integration layer will serve to translate functions and calls from the Beacon v2 implementation into native service calls for RAPTOR*,* allowing RAPTOR to reuse the Beacon v2 reference implementation with minimal modifications. The GA4GH Data Repository Service (DRS)^35^ is a set of APIs providing consumers (both users and workflows) with direct access to data in a repository. A key feature of DRS is the provision of a Universal Resource Identifier (URI) to provide an exclusive identification for a single file or group of files within a repository. This will allow data consumers to access resources without prior knowledge of repository’s data organization or file hierarchy. RAPTOR also enables data contributors to define groups of files or directories termed a Collection*.* RAPTOR Collections can be shared and referenced by data consumers independently of Collection’s parent dataset. Therefore, it may be possible to extend RAPTOR Collections to a DRS-compliant resource*.* DAC approval can be integrated into authentication and authorization workflows before retrieving data using DRS. Destination endpoint IP address for remote retrieval can also be whitelisted.

### Federated analysis with data in place

Even with DRS approval and IP whitelisting, remote data retrieval may inevitably weaken RAPTOR’s safe purpose assurance. In addition, when working with datasets on the scale of multiple terabytes or more, cost and latency for replicating large data volume across networks can quickly become prohibitive. A more feasible approach would be to send the compute job to the data and return outputs of the job. GA4GH has defined three standards for sending compute jobs to be executed on remote sites, including the Tools Registry Service (TRS), Workflow Execution Service (WES), and Task Execution Service (TES).^36^ TRS provides standardization for tools discovery, providing standardized descriptions of docker-based tools and popular workflow engines.^37^ Dockstore (https://dockstore.org/) is an example of a TRS-compliant container repository. WES then builds on top of TRS to provide a common interface to interact with TRS tools and workflows. TES is similar to WES in that it also defines an interface defining and running compute tasks.^38^ TES is differentiated from WES in that a TES task can be modeled as a single job execution such as executing a single script or a command, while a WES is designed for executing a pre-composed workflow. It is possible for a TES to be “nested” within a WES. For example, a Nextflow workflow can serve as a TES client submitting tasks to a TES server.^39^

These three services, however, are insufficient for RAPTOR to provide federation services to third-party repositories*.* As a serverless application, RAPTOR does not have any ready virtual servers to execute remote workflows or tasks requests. The Analytics Workspace is also not suitable as it is designed for direct interactions with end-users and would be unwieldy to automate. In addition, these standards do not provide an easy way to inform RAPTOR of the type of virtual machines required for execution. As an example, while TES allows for resource specification within a task request, the resource request is defined in the form of cores, random access memory (ram), disk size, and name or URI of a docker container. These parameters alone are not sufficient to identify an instance type. Specifically, on AWS, just specifying “2 cores and 8 GB ram” maps to at least 7 different instance types, each with a unique hardware profile, optimized for different use-case. There is also no means of specifying a type of platform (x86 or ARM, Memory-optimized, or graphical process unit-enabled), and loading containers or images from third-party repository is not compatible with RAPTOR’s operations. To preserve RAPTOR’s safe purpose assurance, RAPTOR administrators would have to manually clear an AMI or container before allowing it for use, which would require the container or image to be hosted within a restricted repository trusted by RAPTOR administrators. The remote user would thus have to perform an “out-of-band” communication with the data contributor on RAPTOR to learn the identifier of the AMI or container to be referenced with TES. This may complicate workflow scripting or automation.

To overcome these limitations, RAPTOR’s development team evaluated an early concept where we could predefine and associate an AMI with a dataset. AMIs allow users to define key features for a virtual server, such as platform-configured tools (x86 or ARM, ROCm or CUDA) and even reference files. At the same time, AMIs provide some flexibility for users to determine the appropriate sizing (core counts, amount of ram, volume of attached disks) during runtime. The exchange only involves the universal identifier of the AMI, not the actual image file. When a remote compute request has been received, RAPTOR will initialize a virtual machine using AWS Batch*,* with core count and ram size matching the values from TES. Batch will initialize the virtual machine, execute the command, write back the output to requestor, and terminate the machine.

### Case study: Prototype of federated analysis on RAPTOR

As a case study, the RAPTOR development team recognized the challenge of performing these activities within the defined set of GA4GH APIs. We thus implemented an early-stage proof of concept where a RAPTOR instance received a remote computation request together with an AMI and docker identifier, with task execution and termination of the virtual server after the results were returned.

In the prototype, we evaluated a federated imputation workflow in RAPTOR by performing imputation on a Primary Open Angle Glaucoma (POAG) dataset using Minimac4 (version 1.0.0) with the same SG10K health reference panels. However, in this scenario, two RAPTOR instances were created, one hosting POAG and the other SG10K health panels. Each customized instance had added functions, providing a minimal implementation of TES and task invocation via the API Gateway and Batch*.* The team limited the proof of concept’s scope to TES since the initial DRS exchange only involved inserting additional data content under “alias” section of response to a DRS “Get” call. Hence, we configured the RAPTOR instances assuming that information can be packed within the single DRS get call. Specifically, these include the AMI id, minimac4 imputation commands, mount paths and customized AWS IAM role (role created for this workflow only, will be destroyed after the end of workflow), and egress IP address. The IAM role and IP were used to configure POAG RAPTOR to allow network filesystem (NFS) read-write mounts from the SG10K RAPTOR*.* This allowed SG10K RAPTOR to read and write data to POAG RAPTOR.

**Figure 3:**
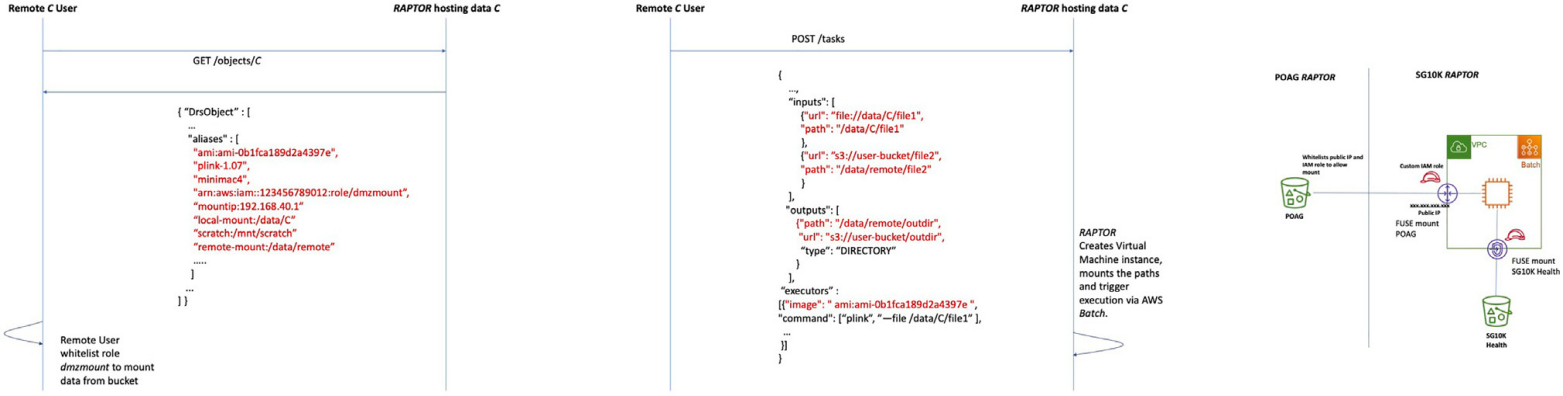
Using existing GA4GH DRS and TES for federated computation on RAPTOR (i) A client invokes Get to describe an existing data collection ‘C’ on RAPTOR. In addition to content and access descriptions, RAPTOR also sends identity of the AMI associated with C under ‘alias’. The provided information includes AMI id, pre-installed tools, mount points for accessing data, and the customized IAM role and endpoint IP; these are used by RAPTOR to read and write data from the remote client. (ii) The remote client invokes a task using TES. In the call, inputs are used for the client to inform RAPTOR which URI is to be mapped to which mount point. Under executors, the remote client will inform RAPTOR which AMI is to be used. (iii) Batch instantiates an EC2 machine AMI and parameters provided by TES task. The machine will mount paths from POAG RAPTOR and SG10K RAPTOR within the same machine using the customized IAM role.

The complete imputation workflow could then be broken down into four main TES tasks: 1) creating unphased variant call format (VCF) files, 2) conversion to phased VCFs, 3) addition of prefix “chr” to entries in phased VCFs, and 4) imputation. During the evaluation, the POAG RAPTOR submitted the four TES tasks creation calls in sequence. Each task was invoked after the previous one had been completed successfully (validated via TES “Get task” call). With every task received, the SG10K RAPTOR will submit a job to AWS Batch service using its account with a predefined Virtual Private Network (VPC) using parameters received from TES calls. To allow reading both datasets during analysis and writing of outputs to either (or both) of RAPTOR instances, a virtual machine was set up to run filesystem in userspace (FUSE) mounts to both POAG RAPTOR and SG10K RAPTOR. AWS Batch service will run the TES tasks and write outputs back into POAG RAPTOR*.* After the end of an AWS Batch job, AWS Batch Service will automatically stop and delete all resources associated with the task (except files written to output directories). Users on POAG RAPTOR can monitor the task status by issuing TES “Get task” calls to SG10K RAPTOR*.* Once a task was completed, the user will submit the subsequent TES task until all 4 tasks are completed (Figure 3).

Using the POC system, we completed the imputation of POAG chr21 with SG10K Health. Results from the POC system were consistent with results from work done on the production RAPTOR platform (whole-genome imputation of POAG with SG10K Health panel). With this approach, the “host” site controls the tools, security policies, and the virtual machines, while the remote user directs where the outputs are written. The use of *Batch* ensures all interim data in scratch will be deleted. Hence, we believe this protype approach can provide both the remote user and the host strong protections against data leaks.

## Discussion

### Value of RAPTOR-like platforms for genomics research

A cornerstone to precision and personalized medicine is the ability to effectively access and thoroughly evaluate a multitude of genomic datasets to improve our molecular understanding of health and disease processes. Managing large-scale data analysis across multiple datasets, however, remains challenging. Hyper-scale computational capabilities provided by CSP such as AWS provide a full suite of computational resources to enable concurrent in-data-analysis data on an AWS S3 Bucket. Nevertheless, harnessing these resources requires knowledge on programming and cloud computing expertise. Additionally, the sensitive nature of genomics data and increased emphasis on data governance and security limit capabilities of individual labs to setup a cloud platform for effective large-scale genomic collaboration studies. Data contributors from public sectors also routinely request for specific controls to be put in place, such as AES256 symmetric for data encryption at rest and the version of TLS/SSH protocols for data encryption during transit. Data hosting facilities may also require certifications such as International Organization for Standardization (ISO) 27001 or multi-tier cloud security (MCTS) Level 3.

RAPTOR provides users with a platform, which addresses these computation and security concerns. Through RAPTOR, users seamlessly run their analysis as they would have on an on-premise system or their private cloud machine while still staying compliant to overarching governance and security regulations. Our case example on performing imputation on large-scale and consortia-level datasets demonstrates these capabilities in RAPTOR and highlights the potential of integrating various genetic resources to improve genetic studies, while, at the same time, remaining compliant to required restrictions of sensitive genetic data.

Beyond security, datasets hosted on RAPTOR become FAIR (Findable, Accessible, Interoperable, and Repeatable).^40^ RAPTOR allows contributors to add metadata as tags to their datasets, allowing for quick way to filter out relevant datasets using RAPTOR’s simple search form (example: Show all datasets where “Country = Singapore and ethnicity = Malay"). RAPTOR users can submit request to access any dataset hosted on RAPTOR, with levels of access determined by the respective DACs. As RAPTOR manages all data provisioning tasks, users can start working on the requested datasets immediately upon receiving approval. RAPTOR’s AMI-sharing feature further allows users to save and share their intermediate data and work environment (i.e., Juypter-style Notebook or even the whole Linux machine) with other RAPTOR users, enabling interoperability and repeatability.

RAPTOR went live in 2021 and has uniquely built a collection of Asian genetic SNP array-based genetic datasets that are primarily from the local Singapore ethnic population groups (Singapore Chinese, Malay, and Indian datasets). As of October 2022, RAPTOR is hosting close to 100,000 samples with more than half from Singapore (Table 2).

**Table 2:** Datasets hosted on RAPTOR. # denotes studies with samples from Singapore

Studies	*N*	Ethnicity	Data available
Singapore Chinese Health Study^#^	23,756	Chinese	GWAS Array/CNV data
SCHS Coronary Artery Disease Cohort^#^	2,003	Chinese	GWAS Array
Singapore Chinese Eye Study^#^	1,889	Chinese	GWAS Array
Singapore Malay Eye Study^#^	2,542	Malay	GWAS Array
Singapore Indian Eye Study^#^	2,538	Indians	GWAS Array
Singapore Coronary Artery Disease Genetics Study^#^	1,943	Chinese, Malay, and Indians	GWAS Array
Study of Macro-angiopathy and Micro-vascular Reactivity in Type 2 Diabetes (SMART2D) dataset^#^	1,893	Chinese, Malay, and Indians	GWAS Array
Diabetic Nephropathy dataset^#^	2,563	Chinese, Malay, and Indians	GWAS Array
SG10K Health r5.5^#^	9,770	Chinese, Malay, and Indians	Imputation Reference Panel
Jerusalem Perinatal study	2,593	Israeli	GWAS Array
Singapore Prospective Study Program^#^	2,434	Chinese	GWAS Array
Primary Open Angle Glaucoma Study^#^	3,580	Chinese	GWAS Array
Singapore Cohort Of the Risk factors for Myopia^#^	1,029	Chinese	GWAS Array
Breast Cancer Association Consortium	40,001	Pan Asian	GWAS Array

### Challenges with scaling up of hosted data

The footprint for next generation sequencing (NGS) data is easily an order of magnitude higher than array data. While we do not foresee major issues to scale up data footprints for RAPTOR’s data management (DynamoDB and Lambda) and bulk storage (S3) services, larger data may pose challenges in staging latency and operating cost.

Several RAPTOR operations require data to be staged across buckets or data stores. In particular, data within a workspace have to be staged from S3 into FSX Luster. FSX only creates file metadata when provisioned, relying on lazy loading as a mechanism for moving data into FSX Luster. Load time is usually limited by S3 read throughput (sustained throughput usually slightly over 100 MBs^-1^), and load time scales linearly with file size. Hence a user working on a sizable collection of whole-genome sequencing data may experience considerably longer delay when performing initial file reads. With larger data footprints, users also likely require a larger scratch (FSX Luster) which has to stay “alive” for a longer duration. Processing large NGS files (such as VCF manipulation, variant calling) would often require machines with high runtime memory (RAM). Such machines would cost more leading to higher runtime costs.

As RAPTOR scales in both number of datasets and its footprint, cost recovery becomes an increasingly important aspect for RAPTOR operations. RAPTOR recovers most of its cost from data users. Data owners pay the minimum amount to partially cover platform overheads, for example, constant security scans running in the background. Conversely, data users will pay for the full cost of resources used, plus a small markup to ensure platform sustainability. This model encourages data owners to host their datasets on RAPTOR as the majority of costs are derived from data usage and not through storage of data.

RAPTOR provides a few tools to help data users manage their costs. A cost dashboard on the user’s landing zone displays user’s cost incurred. Additionally, users have the choice to suspend their workspace when there is a pause in their work. Unlike “terminate”, suspend does not delete the working environment, but the user will not incur cost on virtual machines in suspend mode. In addition, the team will be introducing features to allow users to set email alert once their incurred costs go over defined threshold.

Beyond cost and data latency, onboarding of more datasets may also add to the workload of the operations team. RAPTOR employs a “man-in-the-middle” approach for data security. Activities such as data ingress and AMI import require review and approval by RAPTOR’s administrators. This is one of the contributing factors to RAPTOR adopting a shared responsibility model for data management. The RAPTOR administrators focus on enforcing platform and IT security, while the DAC controls safe use of data. This approach allows RAPTOR administrators to focus on a very defined aspect of data security and compliance and not become overburdened with workload as we scale up the datasets hosted within RAPTOR.

### Risk management

RAPTOR’s internal risk assessment has highlighted two main risks: 1) security breach from malware introduced during data ingress or AMI import and 2) security vulnerabilities or failures within AWS’s native infrastructure. RAPTOR takes two approaches to address the first risk. Firstly, RAPTOR administrators work closely with DAC representatives to ensure they understand how RAPTOR procedures provide data safety. RAPTOR administrators will ensure DAC representatives are trained and are comfortable with using RAPTOR’s controls. Secondly, RAPTOR does not allow direct data ingress or egress. Data must be isolated on a different storage bucket while it is being cleared, thus creating a barrier to prevent malicious actors leveraging egress or ingress reviews to launch attacks. This isolated review mechanism also applies to AMI imports.

To mitigate the second risk, we also adopted a zero-trust system design for RAPTOR. Segregration is applied judiciously with system zero trust between all. RAPTOR is hosted on a dedicated AWS account, with all development and testing occurring in a separate account. All AWS roles for operating RAPTOR are segregated. For instance, a dedicated role is used to deploy RAPTOR services and another for regular operations. All accounts are protected with multi-factor authentication with 3 months password rotation. There are no “super user” accounts that can manage all resources in this AWS account (root account disabled). RAPTOR services are segregated into different virtual networks. All services are guarded via policy, network, and keys. There is no assumed trust between any resource within RAPTOR. All network activity within this account is monitored and logged. Our rationale for the high level of segregation is the underlying assumption that if there is an unknown vulnerability in a key native service, processes must be implemented to limit damage and “blast radius”. In addition, “break-glass” protocols are in place to where administrators can activate kill switches to immediately lock down all assets within RAPTOR.

### Future objectivies for RAPTOR

In the long term, platforms such as RAPTOR are likely to represent options as trusted custodians for national-scale genomic data. RAPTOR’s next phase will also focus on expanding the types of data hosted on the platform. Various transcriptomic datasets from local population datasets are expected to be housed in RAPTOR, facilitating larger-scale transcriptomic studies and combinatorial expression quantitative trail loci studies. To do so, RAPTOR will continuously update security measures and operating procedures to ensure compliance with relevant data and IT governance policies. While we have not extensively reviewed RAPTOR’s alignment to similar policies from other jurisdictions, we note that RAPTOR’s core features facilitate alignment to 5 of the 7 core principles of European Union’s data protection law (GDPR).^41^ These include lawfulness, fairness, and transparency and purpose limitation by providing the DAC with control over how data are used and who can access data, data minimization where data contributors may create cub-collections of data for sharing, integrity and confidentiality through RAPTOR’s security capabilities, and encryption adoptions as well as accountability through the storage of logs of every activity on RAPTOR. Under the shared responsibility model of RAPTOR, data validation and ensuring proper use of data are deferred to the DAC. The DAC reviews all access requests and approves all data ingress and egress. Therefore, principles on accuracy and storage limitations are usually beyond the scope for a platform such as RAPTOR and best specified by the data contributor and DAC.

### Federated analysis and data sharing using GA4GH protocols

For RAPTOR, the ability to integrate with other repositories (either via data movement or federated analysis) will be essential for the next phase of development. GA4GH protocols will be the key interface between RAPTOR and other repositories and platforms. The key challenge, as highlighted, is ensuring this implementation continues while retaining the 5 safes assurances. One current limitation in our study was that, for our DRS implementation, there required be a mandatory, out-of-band approval seeking with dataset’s DAC before a dataset can be retrieved to remote site. Notably, while DRS’s standards require the use of OAuth2 tokens for authentication, the standard is silent on the authentication and authorization procedures. RAPTOR is already utilizing OAuth2 for user authentication. RAPTOR’s authentication mechanism can be extended to applications and scripts (i.e., automated flow triggered by user or software), with IP whitelisting as replacement for 2FA. However, once data have egressed, there would be no effective way for DAC to track subsequent usage or enforce additional compliances. It should, however, be noted that RAPTOR data marked as sensitive (mandatory use of secure analysis workspace) will not be valid for retrieval using DRS.

We propose that RAPTOR’s federated analysis will provide stronger adherence to the 5 safes. The federated analysis environment and tools are pre-determined by the data contributors (i.e., the remote data site) and are immutable. All endpoints are IP-locked, with the pre-determined write out process. The data owners therefore have full control of how the data are to be used and the outputs which will be shared with the remote user. Data access expiry can be automatically enforced by RAPTOR.

RAPTOR’s federated analysis is implemented with GA4GH DRS and TES API interfaces. RAPTOR utilizes free text sections within the DRS and TEST schemas to exchange essential integration information, including the data paths and tools available for use. The goal of using DRS and TES APIs is not to allow “ad-hoc” invocations from remote users. The goal of using GA4GH APIs is mostly to reduce the implementation and customization overheads.

### RAPTOR’s technical advantage and roadmap

A key advantage from building RAPTOR as a “serverless” application is that RAPTOR is completely abstracted from both the system hardware and software, including the operating system. Instead, core RAPTOR services including the user interface and user management are plugged directly into AWS’s hyper-scale compute and storage fabric. All button clicks and function calls are distributed to a managed cluster of servers that ensures consistent performance that scales with load while keeping the cost of upkeep low since RAPTOR is billed only for the resources for the functions executed, not for the number of servers it ran on. Apart from cost efficiency and performance scaling, a serverless resource also provides an easier pathway for integration with existing applications and services. The reason is that major CSP such as AWS reduces entire system infrastructure as a service to a series of functions calls that are by design consistent with cloud-native service calls utilized for core RAPTOR functions. This means it is programmatically similar for us to deploy a complete system of applications as to implement a button on the user interface, hence appreciably reducing the effort and time required to integrate third-party applications. For example, we have plans to enhance hosted data’s findability by providing improvements on metadata capture and adopting GA4GH’s Data Use Ontology (DUO).^42^ However, instead of building more native services, we are evaluating ways to integrate with third-party tool suites such as those from Center for Expanded Data Annotation and Retrieval (CEDAR).^43^ We will package the entire system into a software-defined infrastructure that will be provisioned on demand.

Based from learnings from this prototype, the next developmental goal for RAPTOR is put to implement a feature of complete data exchange with third-party data sources (data federation) using combinations of community defined standards, including GA4GH Beacon v2, DRS, and WES.

In conclusion, we propose that the RAPTOR computational platform provides researchers a significant resource that enables power of cloud-based computing while ensuring a safe and secure environment to meet regulatory requirements for genomic data. Additionally, RAPTOR enables flexibility in data federation and offers potentials to integrate multiple data repositories that would enable for more effective analysis of large-scale genomic data.

### Limitations of the study

One limitation of this study is that it reviews approaches to security but not implementation of controls in details which is also important for scaling up and user acceptance. A second limitation is that we did not explore effectiveness of alternate emerging security approaches in complying with relevant security policies, such as privacy-preserving analytics.

## STAR★Methods

### Key resources table

**Table:** 

REAGENT or RESOURCE	SOURCE	IDENTIFIER
**Software and algorithms**		

Minimac4 (version 1.0.0)	Howie et al.^44^	
Plink (version 1.9)	Chang et al.^45^ PMID: 25722852; PMCID: PMC4342193.	

**Other**		

RAPTOR Source Codes	Please reach out to lead contact	
NIH NCAT N3C	https://ncats.nih.gov/n3c/resources	
NGHRI AnVIL	https://anvilproject.org/	

### Resource availability

#### Lead contact

Further information and requests for resources should be directed to and will be fulfilled by the Lead Contact, Shih Chih Chuan at shihcc@gis.a-star.edu.sg.

#### Materials availability

This study did not generate new unique reagents and datasets.

## Data Availability

• RAPTOR does not have an additional Data Access Committee for hosted data. A key design decision for RAPTOR is to provide data owners the ability to directly manage access to their data. A RAPTOR workgroup is in-place to work directly with the owners to facilitate data management process and RAPTOR will forward any access request to the relevant DAC of each study hosted in the platform. All data reported in this paper will be shared by the lead contact upon request. • This paper does not report original code due to the possibility of inadvertently revealing security vulnerabilities. However, desensitized code can be provided upon contact with reasonable request. • Any additional information required to reanalyse data reported in this paper is available from lead contact upon request.
