# Supplementary material for: A five-safes approach to a secure and scalable genomics data repository
Source: iScience. 2023 Mar 31;26(4):106546. doi: 10.1016/j.isci.2023.106546 (PMC10139958; doi:10.1016/j.isci.2023.106546)
Supplement: Document S1. Figure S1 and Table S1 [file mmc1.pdf]

iScience, Volume *26*

Supplemental information

A five-safes approach to a secure and scalable genomics data repository

Chih Chuan Shih, Jieqi Chen, Ai Shan Lee, Nicolas Bertin, Maxime Hebrard, Chiea Chuen Khor, Zheng Li, Joanna Hui Juan Tan, Wee Yang Meah, Su Qin Peh, Shi Qi Mok, Kar Seng Sim, Jianjun Liu, Ling Wang, Eleanor Wong, Jingmei Li, Aung Tin, Ching-Yu Cheng, Chew-Kiat Heng, Jian-Min Yuan, Woon-Puay Koh, Seang Mei Saw, Yechiel Friedlander, Xueling Sim, Jin Fang Chai, Yap Seng Chong, Sonia Davila, Liuh Ling Goh, Eng Sing Lee, Tien Yin Wong, Neerja Karnani, Khai Pang Leong, Khung Keong Yeo, John C. Chambers, Su Chi Lim, Rick Siow Mong Goh, Patrick Tan, and Rajkumar Dorajoo

Supplemental information

RAPTOR: A Five-Safes approach to a secure, cloud native and serverless genomics data repository.

Chih Chuan Shih, Jieqi Chen, Ai Shan Lee, Nicolas Bertin, Maxime Hebrard, Chiea Chuen Khor, Zheng Li, Joanna Hui Juan Tan, Wee Yang Meah, Su Qin Peh, Shi Qi Mok, Kar Seng Sim, Jianjun Liu, Ling Wang, Eleanor Wong, Jingmei Li, Aung Tin, Ching-Yu Cheng, Chew-Kiat Heng, Jian-Min Yuan , Woon-Puay Koh, Seang Mei Saw, Yechiel Friedlander, Xueling Sim, Jin Fang Chai, Yap Seng Chong, Sonia Davila, Liuh Ling Goh, Eng Sing Lee, Tien Yin Wong, Neerja Karnani, Khai Pang Leong, Khung Keong Yeo, John C Chambers, Su Chi Lim, Rick Siow Mong Goh, Patrick Tan and Rajkumar Dorajoo

Supplementary Information This pdf includes

Table S1 and Figure S1

	NCATS N3C	GIS RAPTOR	NHGRI AnVIL
**Restrictions on data analysis**	Secure analysis in place, with predefined tools only.	Secure analysis in place.<br><br>Has functions for users to compose custom AMI and submit to data set's DAC for approval	Secure analysis in place and ability to export data to external tools (e.g. Terra).<br><br>Provides native integration with large collection of community-based tools including Docker Hub and GALAXY
**Downloading of hosted data sets**	No	DAC for each data set to decide.	Downloads available for selected datasets
**Approval for user access request**	N3C DAC	No RAPTOR DAC.<br><br>DAC of individual data sets will act on request from RAPTOR's console	AnVIL DAC

Table S1: Three key data security differentiators between RAPTOR and NCAT N3C data enclave and NHGRI AnVIL. Related to STAR Methods

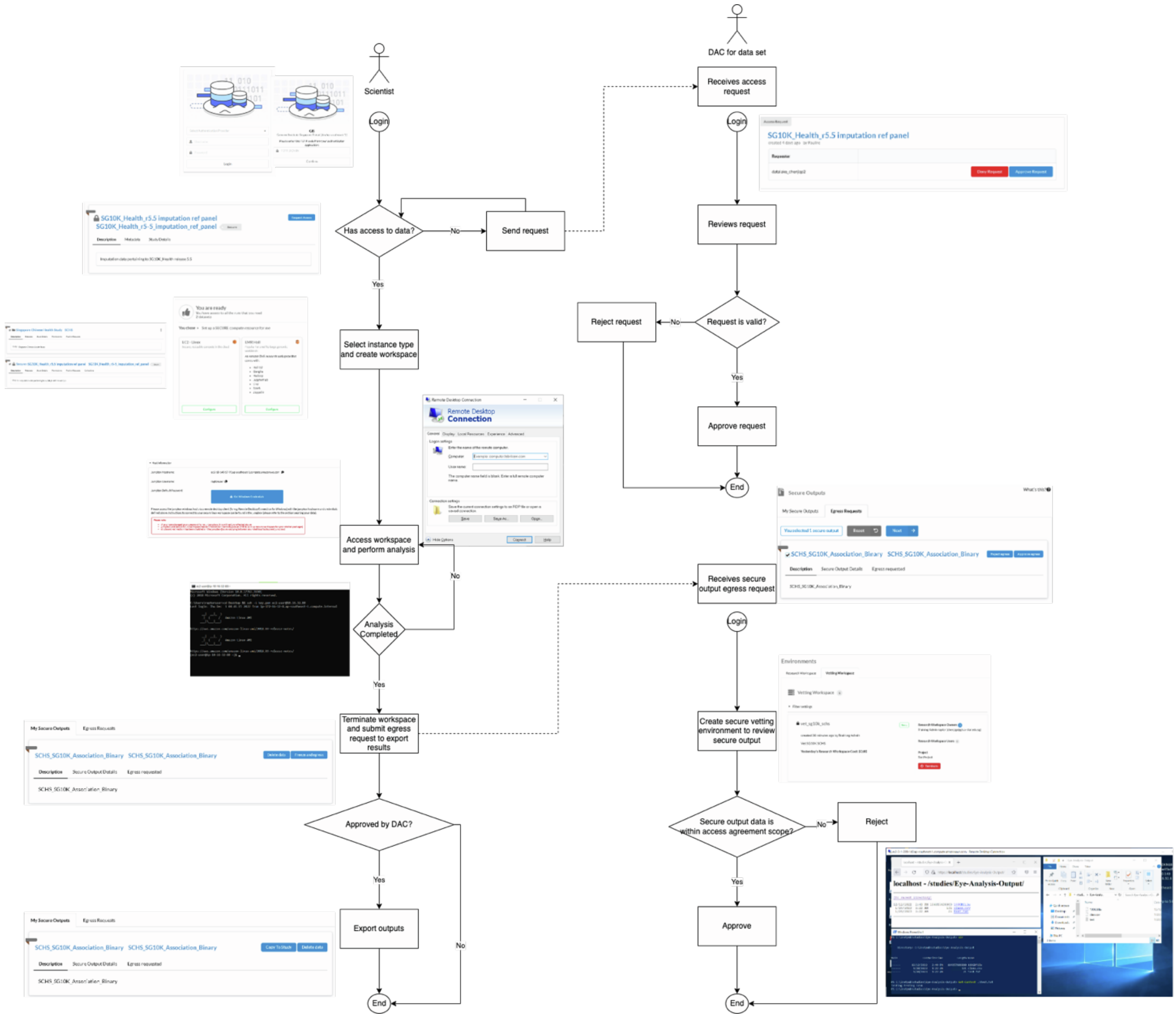

Figure S1: Flow chart detailing sequences of action for how individual components within RAPTOR comes together in the context of Secure Imputation Analysis case study. Related to figure 2

*Egress review utilises a read-only workspace. Within the review workspace, reviewers can access the data for egress using regular file explorer or command line
